# Validation and application of a novel cholesterol efflux assay using immobilized liposomes as a substitute for cultured cells

**DOI:** 10.1042/BSR20180144

**Published:** 2018-04-13

**Authors:** Yuna Horiuchi, Shao-Jui Lai, Azusa Yamazaki, Ayaka Nakamura, Ryunosuke Ohkawa, Kouji Yano, Takahiro Kameda, Shigeo Okubo, Shitsuko Shimano, Michio Hagihara, Shuji Tohda, Minoru Tozuka

**Affiliations:** 1 Analytical Laboratory Chemistry, Graduate School of Health Care Sciences, Tokyo Medical and Dental University (TMDU), 1-5-45 Yushima, Bunkyo-ku, Tokyo 113-8519, Japan; 2 Center for Genomic and Regenerative Medicine, Graduate School of Medicine, Juntendo University, 2-1-1 Hongo, Bunkyo-ku, Tokyo 113-8421, Japan; 3 Department of Medical Technology, School of Health Sciences, Tokyo University of Technology, 5-23-22 Nishikamata, Ota-ku, Tokyo 144-8535, Japan; 4 Faculty of Health Science Technology, Bunkyo Gakuin University, 2-4-1 Mukogaoka, Bunkyo-ku, Tokyo 113-8668, Japan; 5 Clinical Laboratory, Medical Hospital, Tokyo Medical and Dental University (TMDU), 1-5-45 Yushima, Bunkyo-ku, Tokyo 113-8519, Japan

**Keywords:** cholesterol efflux, cardiovascular disease, fluorescently labelled cholesterol, high density lipoprotein, polyethylene glycol, reverse cholesterol transport

## Abstract

Estimation of the function as well as the amount of high-density lipoprotein (HDL) is required to predict the risk of cardiovascular disease development. Cholesterol efflux capacity (CEC) is the key metric for determining the antiatherosclerotic function of HDL. However, the assay methods currently used to calculate CEC are not ideal for clinical use as they require the culture of cells. In the present study, we developed a novel CEC assay using immobilized liposome-bound gel beads (ILGs), containing fluorescently labeled cholesterol, as a substitute for cultured cells. When apolipoprotein B-100 depleted serum, obtained by polyethylene glycol precipitation, was used as the cholesterol acceptors, the basic properties of this method, such as the available range of HDL-cholesterol, efflux temperature and time, and normalization parameters, indicate that this method is sufficient to estimate CEC. Furthermore, the CEC values obtained with this ILG method were also correlated with those obtained with a conventional method using THP-1 macrophages derived foam cells and ^3^H-cholesterol as a tracer (*r* = 0.932). Overall, this novel cholesterol efflux assay method is a realistic and effective alternative to current methods in the field while also being easier to use in clinical laboratories as neither cell culture, radioisotope nor ultracentrifugation is required.

## Introduction

It is widely known that high-density lipoprotein (HDL) has an antiatherosclerotic function, and an increase in HDL may prevent the development of atherosclerotic diseases, including cardiovascular disease (CVD) [1,2]. Thus, drugs that increase HDL-cholesterol (HDL-C) levels have attracted a great deal of attention. Unfortunately, while many of these drugs do mediate in increase in HDL-C, none of the current treatments have reached their expected efficacy, suggesting that not only the amount of HDL-C, but also its functionality must be considered in estimating atheroprotective ability. HDL has many functions and can act as a reverse cholesterol transporter (RCT) [3] as well as an antioxidant [4], anti-inflammatory [5], antithrombotic [6], and anti-infectious [7] agent. In RCT, cholesterol transport from peripheral tissues, including atherosclerotic lesions, to the liver is recognized as the most important function of HDL [3]. As cholesterol efflux from cells accumulating excess cholesterol is the first step of RCT, this stage is likely when HDL modulates its atheroprotective effects [8]. Indeed, it has been reported that cardiovascular risk can be reduced by up to 67% in the highest quartile of cholesterol efflux capacity (CEC) compared with the lowest quartile [9]. However, the methodologies used to measure cardiovascular health vary widely, making comparisons between studies difficult and limiting their impact [10].

At present, many clinical laboratories measure HDL-C as well as low-density lipoprotein cholesterol (LDL-C) levels to estimate cardiovascular risk. On the other hand, recent research suggests that it is necessary to determine CEC of HDL for a more reliable assessment of cardiovascular risk [11]. Most CEC assay methods require cells, radioisotope-labeled cholesterol as a tracer, and HDL as a cholesterol acceptor [12]. Recently, a simple method was reported using fluorescently labeled cholesterol instead of radioisotope-labeled cholesterol and apolipoprotein B-100 depleted serum (BDS) obtained by polyethylene glycol (PEG) treatment rather than HDL obtained by ultracentrifugation [13]. However, all of the current CEC assays require the use of cells, the isolation and manipulation of which are often complicated and lengthy, making it difficult for some clinical laboratories to carry out these assays. The development of a simple procedure using a proxy for these cells, thus making it accessible to clinical laboratories, would be invaluable.

In the present study, we developed a convenient CEC assay method using liposomes containing fluorescently labeled cholesterol (BODIPY-cholesterol) as artificial cells. The technique also involves use of resin for size-exclusion chromatography (Sephacryl S-300) for liposome immobilization. Furthermore, we also compared this technique with an established method using foam cells derived from THP-1 cells (a human monocytic cell line) with ^3^H-cholesterol as the tracer.

## Materials and methods

### Chemicals

All chemicals were purchased from Wako Pure Chemical Industries (Tokyo, Japan) if not stated otherwise.

### Serum samples

Serum samples from 23 patients with varying HDL-C levels were obtained from the Clinical Laboratory of Medical Hospital at the Tokyo Medical and Dental University. Normal serum samples were obtained from apparently healthy eight volunteers at the Graduate School of Health Care Sciences, Tokyo Medical and Dental University. In both laboratories, 9 ml of blood samples were drawn into serum separator-containing tubes (TERUMO, Japan), which were then centrifuged at room temperature for 15 min. Serum samples (1.0–1.5 ml) from the patients were aliquoted into 2.0 ml of Eppendorf tubes after the routine examinations and stored at −80°C until use. The study was approved by the ethics committee of the Faculty of Medicine, Tokyo Medical and Dental University, a common committee to both laboratories, and performed from June to November in 2017.

### Measurement of serum lipids

Total cholesterol (TC), triglyceride (TG), LDL-C, and HDL-C were measured using enzymatic test kits (Kyowa Medex Co., Japan).

### Cell culture

Cell culture was carried out as described previously [12]. Briefly, THP-1 cells were maintained in RPMI-1640 media (Sigma-Aldrich) containing 10% fetal bovine serum (FBS), 0.1% penicillin/streptomycin, and 0.1% nonessential amino acids.

### Preparation of immobilized liposome-bound gel beads (ILGs)

ILGs were prepared as previously described [14,15] with some modifications. Briefly, egg lecithin (106 mg) and cholesterol (23 mg) were dissolved in 6 ml of chloroform, and 300 μl of 0.5 mM 4,4-difluoro-4-bora-3a,4a-s-indacene labeled cholesterol (BODIPY-cholesterol; Avanti Polar Lipids, AL) in ethanol was added to the solution. The lipid film, formed under N_2_ gas, was then resolved in ether, and the solvent was removed by evaporation. After performing this step twice, the lipid film was completely dried under N_2_ gas and suspended with 7 ml of 10 mM Tris-HCl (pH 7.4) containing 150 mM NaCl and 1 mM Na_2_EDTA (Buffer A). Dried Sephacryl S-300 gel beads (1 g; GE-Healthcare Japan) were then added to the liposome suspension followed by swelling for 30 min at room temperature (RT). The mixture was then treated by seven cycles of freezing (−80°C) and thawing (in water at RT) to induce the formation of large multilamellar vesicles in the Sephacryl S-300 beads [15]. Finally, the gel was sufficiently washed with Buffer A, centrifuged, and resuspended with 5 ml of Buffer A. The gel suspension was stored in the dark at 4°C.

### Preparation of HDL and apolipoprotein B-100 depleted serum (BDS)

HDLs (1.063–1.21 g/ml) were isolated from serum obtained from the patients and healthy subjects by ultracentrifugation as described previously [16]. The HDL fractions were then dialyzed with PBS. BDS was prepared as described previously [13]. Briefly, 40 μl of 20% polyethylene glycol 6000 (PEG) in 200 mM glycine buffer (pH 7.4) was added to 100 μl of serum. After vigorous mixing and incubation at RT for 30 min, the mixture was centrifuged at 15000 rpm for 30 min. The supernatant was isolated and defined as BDS.

### Cholesterol efflux assay using ILGs (ILG/BODIPY method)

The ILG was uniformly suspended and an aliquot (100 μl) was immediately pipetted into a 2 ml of Eppendorf tube. The necessary number of tubes for replicate analyses was prepared at the same time to avoid any significant variation in the amount of ILG. Cholesterol acceptor solution (150 μl), in this case HDL, BDS (3.33% concentration as a serum), or Buffer A (as a control), was then added to the ILG followed by incubation in the dark at RT for 16 h if not stated otherwise. The BDS was used at a final concentration of 2% serum as described previously [13]. The mixture was then resuspended by vortexing and centrifugation. The supernatant (100 μl) was transferred into a 0.5 ml of Eppendorf tube and centrifuged again to remove residual gel beads. The supernatant (75 μl) was then moved into a 96-well plate and the fluorescence was measured (*E*_x_: 485 nm, *E*_m_: 538 nm). The fluorescence intensity of the supernatant corresponds to the CEC, which is reported for each sample as arbitrary units. All samples were assayed in triplicate.

### Cholesterol efflux assay using THP-1 cells (THP-1/^3^H-cholesterol method)

A cholesterol efflux assay using THP-1 cells was carried out as described previously [12]. Briefly, THP-1 cells (2.5 × 10^5^ cells/well) were differentiated into macrophages via culture in RPMI-1640 medium containing 100 ng/ml of phorbol 12-myristate 13-acetate (PMA; Sigma-Aldrich) supplemented with 0.2% bovine serum albumin (BSA) for 2 days. Culture medium was then changed to RPMI-1640 containing acetylated LDLs (acLDLs) (50 μg of protein/ml), T0901317 (1 mmol/l; Enzo Life Sciences), a liver X receptor (LXR) agonist for promoting expression of ABCA1, ^3^H-cholesterol (1 μCi/ml; PerkinElmer), and BSA (0.2%). In doing so, the THP-1 macrophages were converted to foam cells. After equilibration with RPMI-1640 supplemented with T0901317 (1 mmol/l) and BSA (0.2%) for 18 h, the cells were incubated with BDS or HDL in RPMI-1640 for 4 h. CEC was calculated as the percentage of radioactivity in the medium as per the following formula: {^3^H-cholesterol in medium/(^3^H-cholesterol in medium + ^3^H-cholesterol in cells)} × 100 − the percentage of passive diffusion in the case of no cholesterol acceptor. All samples were assayed in triplicate.

### Statistical analysis

Data are presented as the mean ± SD. We used Student’s *t*-test (unpaired, 2-tail) to determine statistical significance between the mean CECs in triplicate assays using the washing and no washing ILGs. A *P* value less than 0.05 was considered statistically significant. Coefficient of variation (CV%) is the percentage of SD against the mean value.

## Results

### Stability of the ILG

**Figure 1:**
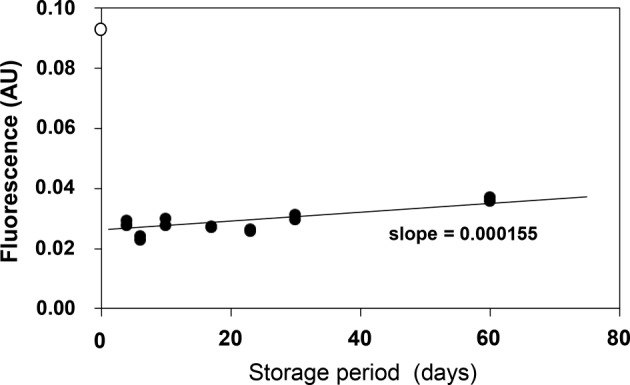
Stability of immobilized liposomes in Sephacryl S-300 gel (ILG) For this cholesterol efflux assay, 100 μl of ILG suspension was mixed with 150 μl of Buffer A in multiple tubes and kept in the dark at 4°C. At various time points (0, 2, 4, 6, 10, 17, 23, 30, and 60 days), 90 μl of supernatant from three tubes was divided into new tubes and kept in the dark at 4°C. After 60 days in storage, the fluorescence (*E*_m_: 485 nm, *E*_x_: 538 nm) of 75 μl of the supernatant was simultaneously measured in triplicate. White circles indicate the CEC of 50 μg protein/ml HDL at the start of experiment; AU, arbitrary unit.

To estimate stability, the ILG (divided into Eppendorf tubes) was kept in the dark at 4°C. The fluorescence of the supernatant increased from 0.0271 arbitrary units on day 0 to 0.0304 on day 30 and then up to 0.0365 after 60 days in storage (Figure 1). When this lot of ILG was used for the efflux assay at day 0, the CEC of 50 μg of protein/ml HDL was 0.0941 arbitrary units.

### Reproducibility

The CEC was measured for two serum samples ten times each to evaluate reproducibility within a sample during analysis (Table 1). The reproducibility for the assay was within an acceptable CV range (5.0%).

**Table 1:** Reproducibility of CEC assay

	CEC (AU) (mean ± SD)	CV (%)
Sample 1	0.0801 ± 0.0040	5.0
Sample 2	0.1088 ± 0.0036	3.3

Abbreviation: AU, arbitrary unit.

### Effect of incubation temperature on CEC

**Figure 2:**
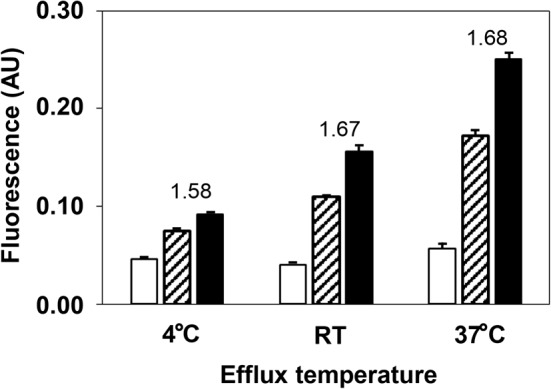
Effect of efflux temperature on CEC Two serum samples with lower CEC (hatched bar) and higher CEC (closed bar) and Buffer A (open bar) were treated with PEG to prepare the BDS samples. The CECs of these samples were determined at 4°C, RT, and 37°C. The number in the figure represents the CEC ratio (after subtracting base fluorescence) of higher CEC to lower CEC in each sample. All values are reported as the mean + SD (*n*=3); AU, arbitrary unit.

The CEC of two serum samples with different CEC levels were determined at 4°C, RT, and 37°C (Figure 2). Although the CEC of each BDS increased in a temperature-dependent manner, the ratio of CEC (after subtracting base fluorescence) for the serum sample with higher CEC level to that with lower CEC level were almost same between RT and 37°C, but slightly lower at 4°C (1.67, 1.68, and 1.58 respectively). Therefore, subsequent experiments were carried out at RT since a proportional result to CEC at 37°C was obtained with a relatively small variation.

### Effect of ILG washing on CEC

**Figure 3:**
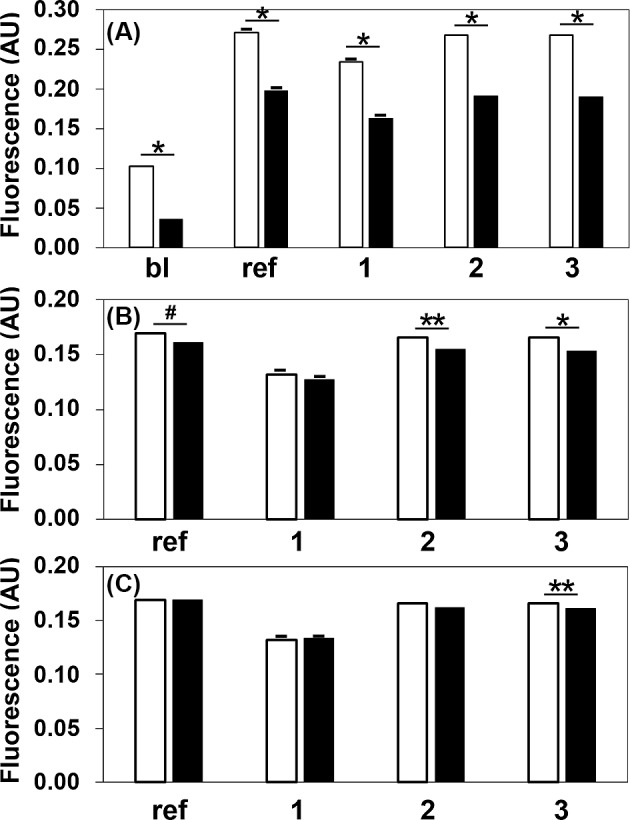
Effect of ILG washing on CEC The CECs of the reference serum (ref), three serum samples (1, 2, and 3), and Buffer A (bl) were determined using the same lot of ILG with (black bar) or without (white bar) washing just before the assays (**A**). The value of control (basic diffusion) was respectively subtracted from each experimental value (**B**). Furthermore, the data were normalized to the CEC of the reference serum, which was set at 1.0 (**C**). The values are reported as the mean + SD (*n*=3). Student’s *t*-test (unpaired, 2-tail) was used for comparison between both means in each sample; *P<0.001, **P<0.01, ^#^*P*<0.05; AU, arbitrary unit; bl, blank; ref, serum defined as the reference.

To clarify the effects of the BODIPY-cholesterol not combined with ILG (background fluorescence) on our CEC assay, the analysis was also performed using ILG with or without washing with Buffer A just before the assay. Although significant differences (*P*<0.001; *n*=3 for each) were observed between the absolute fluorescence measurements of the washing and no washing ILGs (Figure 3A), these differences were extremely reduced when the background fluorescence of the blank was compensated for (Figure 3B). In addition, normalization using one serum sample as the reference also showed no significant differences between the samples, with the exception of one serum sample that had also apparently reduced the differences (Figure 3C).

### Basic properties of the ILG/BODIPY method

**Figure 4:**
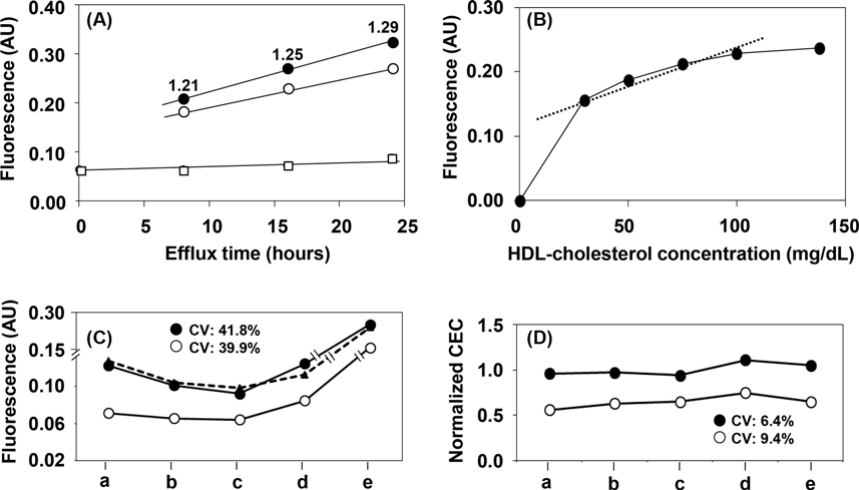
Basic properties of the ILG/BODIPY method To study the basic properties of our novel CEC assay, 75 μl of serum (HDL-C; 40 mg/dl) was mixed with 25 μl of Buffer A or adequately diluted HDL to prepare the five serum samples corresponding to 30 (Buffer A), 50, 75, 100, and 138 mg/dl of HDL-C. Two serum samples corresponding to 50 (white circle) and 100 (black circle) mg/dl of HDL-C and Buffer A were treated with PEG to prepare the BDS samples and the control sample (white square) respectively. The CECs of these samples (final 2% concentration) were determined at 0, 8, 16, and 24 h (**A**). The number in the figure represents the CEC ratio (after subtracting base fluorescence) of 100 to 50 mg/dl HDL-C in each sample. To study the effect of HDL concentration on our novel CEC assay, BDS samples were prepared from five serum samples and Buffer A as described above. The CECs of these samples (final 2% concentration) were determined at 16 h (**B**). To study the effect of reference serum normalization, CECs of the reference serum (dotted line) and two untreated serum samples (white and black circles) were determined on different days with different ILG lots (**C**). The data were normalized to the CEC of a reference serum, which was set at 1.0 (**D**). “a” and “b”: ILG (Lot. No.1) stored for 4 and 17 days respectively; “c” and “d”: ILG (Lot. No.2) stored for 5 and 21 days respectively; “e”: ILG (Lot. No.3) stored for 1 day. All values are reported as the mean (*n*=3), as the SD of the triplicate assays for each sample is too small to indicate; AU, arbitrary unit.

The serum with 40 mg/dl of HDL-C (75 μl) was mixed with 25 μl of adequately diluted HDL to prepare five serum samples corresponding to 30, 50, 75, 100, and 138 mg/dl of HDL-C. The CECs of the two samples with 50 and 100 mg/dl of HDL-C were determined at 0, 8, 16, and 24 h of efflux (Figure 4A). Although the CEC of each BDS increased in a time-dependent manner, the ratio of CEC (after subtracting base fluorescence) for the prepared 100 mg/dl of HDL-C serum sample to that of the 50 mg/dl of HDL-C was almost constant at each efflux time (1.21, 1.25, and 1.29 respectively). The effect of HDL-C concentration on the CEC of the BDS was determined using all five serum samples at the 16-h time point. Interestingly, the CEC was increased for the 30 mg/dl HDL-C sample compared with lower concentrations and gradually increased in a HDL-C concentration-dependent manner (Figure 4B). Although the extent of this increase was not consistent with the corresponding increase in HDL-C concentration, the ratio between these increases was roughly constant in the range from 30 to 100 mg/dl of HDL-C.

To clarify the validity of PEG treatment in the prepared serum samples, the cholesterol concentration of BDS was measured and compared with the HDL-C concentration in each serum sample. The cholesterol levels of the five BDS samples (21.0, 39.5, 57.8, 77.8, and 100.0 mg/dl respectively) were very similar to the theoretical values (21.4, 35.7, 53.6, 71.4, and 98.6 mg/dl respectively) calculated using the dilution rate (serum/PEG = 1/0.4).

In order to reduce the differences among the assays, it is necessary to test and normalize the method using a reference serum. The absolute cholesterol efflux values of the reference serum and two serum samples stored at −80°C showed large fluctuations depending on the assay, ILG sample lot, and storage periods, with a CV greater than 39% (Figure 4C). However, when the test samples were normalized to the reference serum, the variation among the assays was decreased, with a CV less than 10% (Figure 4D).

### Correlations of CECs between the ILG/BODIPY and THP-1/^3^H-cholesterol methods

**Figure 5:**
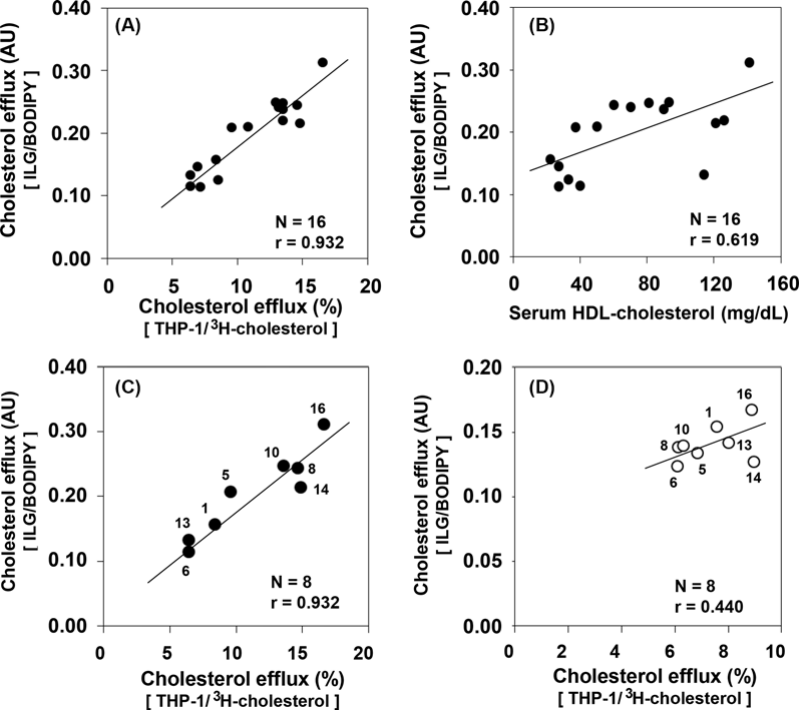
Correlations of CECs between the ILG/BODIPY and THP-1/^3^H-cholesterol methods The CECs of 16 serum samples with varying HDL-C concentrations (22–141 mg/dl) were determined by both the present method (ILG/BODIPY) and a commonly utilized method using foam cells derived from THP-1 cells with ^3^H-cholesterol as the tracer (THP-1/^3^H-cholesterol). Correlation of CECs between both methods was estimated in all samples (**A**). Correlation with CECs obtained by ILG/BODIPY methods and HDL-C was also estimated (**B**). HDLs were isolated by ultracentrifugation for 8 of the 16 samples described above. The data highlighting the correlation between the CEC values obtained with the ILG/BODIPY and THP-1/^3^H-cholesterol methods using BDS as the cholesterol acceptor for 8 samples were transferred from (A) (**C**). The correlation of the CECs using HDL as the cholesterol acceptor between the ILG/BODIPY method (40 μg protein/ml HDL) and THP-1/^3^H-cholesterol method (20 μg protein/ml HDL) was estimated (**D**). The numbers in (C) and (D) correspond to the sample numbers indicated in Table 2; AU, arbitrary unit.

The CECs of 16 serum samples with varying HDL-C concentration (22–141 mg/dl) (Table 2) were determined using both the present method (ILG/BODIPY) and the method using foam cells derived from THP-1 cells with ^3^H-cholesterol as a tracer (THP-1/^3^H-cholesterol). Both methods using BDS as the cholesterol acceptors were strongly correlated (correlation coefficient: 0.932) (Figure 5A). The CECs for 16 serum samples obtained with our ILG/BODIPY method were then compared with their serum HDL-C levels. Our results indicate a relatively strong correlation (correlation coefficient: 0.619) (Figure 5B). Furthermore, the CECs were also determined using isolated HDL as the cholesterol acceptor for 8 of the 16 serum samples. In doing so, we confirmed that the correlation coefficient for the 8 samples did not change significantly compared with that for all 16 samples (Figure 5C). On the other hand, the CECs using HDLs as the cholesterol acceptor were only weakly correlated (correlation coefficient: 0.440) between the two methods (Figure 5D).

**Table 2:** Lipid profiles of the subjects in the correlation assay

Number	TG	TC	HDL-C	LDL-C
1	113	99	22	46
2	–	106	27	61
3	62	117	27	–
4	272	188	33	115
5	276	143	37	74
6	118	–	40	87
7	124	192	50	124
8	154	–	60	68
9	68	131	70	–
10	88	224	81	128
11	70	226	90	126
12	109	231	93	125
13	74	–	114	148
14	67	–-	121	191
15	–	–	126	113
16	123	244	141	89

(mg/dl)

Abbreviations: HDL-C, HDL-cholesterol; LDL-C, LDL-cholesterol; TC, total cholesterol; TG, triglyceride.

## Discussion

Monitoring CEC of HDL has recently been identified as an important method for evaluating cardiovascular health. Unfortunately, the methods currently in use to assay CEC require cell cultures that are time consuming and complex, making their application unrealistic in some clinical settings [10,17]. To circumvent this issue, we developed a novel cholesterol efflux assay method using immobilized liposomes as a substitute for cells. Our results indicate that complicated cell culture is unnecessary.

Liposomes, including egg lecithin, cholesterol, and BODIPY-cholesterol, which were immobilized in Sephacryl S-300 resin, were stable and only slight diffusion was observed over 60 days in storage. Considering the absolute values and the satisfactory reproducibility of supernatant fluorescence, we decided to estimate CEC using the intensity of this fluorescence, rather than the percentage of released BODIPY-cholesterol, which is the typical metric used in other methods. It is important to note that our initial analysis indicated variation in ILG fluorescence which was shown to be largely related to an artifact of resin particles.

It was required to investigate the effect of efflux temperature on CEC measured by our method, since the lipid mobility within ILG could dramatically vary depending on temperature, like that on the cell membrane [18]. Although absolute CEC levels were increased in a temperature-dependent manner, the ratio of CECs obtained from two serum samples was almost constant at RT and 37°C, but slightly lower at 4°C. It means that either of temperature, RT or 37°C, could give us equivalent data in comparison of CECs among many serum samples. Moreover, CECs obtained at 4°C would be available except the defect in low sensitivity, suggesting that this method is possible to use at a wide range of temperature. Therefore, we decided to measure CEC at RT because of relatively small variations with sufficient sensitivity and unnecessariness of any equipment to keep a constant temperature.

Furthermore, it was essential to evaluate the effects of the background fluorescence on CEC value. Although the absolute fluorescence intensity was significantly reduced by ILG washing, the differences between the fluorescence values for each sample were almost completely eliminated by subtracting the blank value. These data indicate that the background (originally releasing BODIPY-cholesterol) does not affect CEC value. Moreover, the possibility was also suggested that slight differences after subtracting each blank value would be further compensated for by normalizing each sample to a reference serum. In doing so, the reproducibility in the assay was shown to be very high, and only one sample was significantly different (*P*<0.05) after normalization. This difference would be caused by a remarkably small variation in each triplicate assay (CV; 0.22% and 0.42%).

To further establish the assay conditions, the effects of efflux time and serum HDL-C concentration on CEC value were estimated. The CEC of two serum samples with HDL-C concentrations of 50 and 100 mg/dl increased in an efflux time-dependent manner in an almost parallel fashion. Although the CEC obtained by this method is not quantitatively parallel to HDL-C concentration, a common point between this method and standard techniques [10,19], this relative estimation would be enough to evaluate the CEC as the function of HDL in a clinical setting. The ratio of CEC between the two serum samples also showed a tendency to increase; however, we believe this would not greatly affect CEC as a fixed efflux time would be used for the assay. Furthermore, the CECs of serum samples with various HDL-C levels (30, 50, 75, 100, and 138 mg/dl) were also determined and the assay appears to be accurate in the 30–100 mg/dl range. Similar to our results described above, this analysis indicates that this method is accurate enough to estimate the CECs of HDL. It is also important to note that in these experiments we prepared these serum samples according to protocols using a mixture of serum with low HDL-C level and adequately diluted HDL obtained by ultracentrifugation. We also confirmed that these prepared serum samples as well as non-diluted serum prepared via PEG precipitation can both be analyzed with this CEC method.

Although the ILG was prepared in line with the fixed procedure described in the “Materials and methods”, differences among gel lots are naturally expected. Repeatedly assaying the CEC of a serum sample using different ILG lots and different storage periods showed large variation, especially when an excess amount of BODIPY-cholesterol was deliberately added during ILG preparation. However, using a reference serum enabled these variations to be minimized, suggesting that we can directly compare the CEC data obtained from different assays without worrying about the ILG lot or storage period so long as a reference serum is also analyzed and used for normalization.

One of the most important observations was that the present method is correlated to the standard methods used currently in the field. Indeed, when BDS was used as the cholesterol acceptor, a correlation was observed between the present method and a common technique utilizing foam cells derived from THP-1 with ^3^H-cholesterol as the tracer. We believe one reason the results of these assays show such a strong correlation is that serum samples with various HDL-C levels (22–141 mg/dl) were used. It is generally acceptable that BDS obtained from serum with high HDL-C levels would be related to a high CEC because of the high concentration of HDL in BDS. In contrast, only a weak correlation was observed when a constant amount of HDL protein was used as the cholesterol acceptor. In this experiment, the CEC reflects the cholesterol efflux ability per unit mass of HDL taking no account of HDL-C level. Further, if a constant cholesterol amount of HDL was used for the efflux study, a different correlation level would likely be observed. Notably, the constant amount of HDL protein used in our experiments was based on the general experimental procedures reported previously [20–22].

As HDL concentration in BDS might be theoretically parallel to HDL-C level in serum, the correlation between the CECs obtained by the present method and HDL-C level was estimated. A relatively good correlation was observed, indicating that CEC is mainly decided by HDL concentration. This contrasts with a previous study that reported no significant correlation between CEC and HDL-C levels [9]. This discrepancy may be due to the relatively narrow HDL-C range being used in the previous study. Furthermore, the positions of some data points on the correlation diagram in the present study did not always reflect the parallel relationship between CEC and HDL-C. This could mean that the BDS isolation method using PEG could vary with regards to HDL recovery and may introduce other proteins whose amount and composition differ among the subjects. This theory is supported by previous reports describing the effect of serum proteins, especially albumin, on CEC obtained using BDS as the cholesterol acceptor [23–25].

In conclusion, the novel CEC method developed here provides satisfactory values which reflect the results obtained from other standard methods in the field and is sufficiently simple to utilize not only in large-scale studies but also in clinical laboratories as an ordinary examination as neither cell culture, radioisotope nor ultracentrifugation is required. While the propriety of BDS as a cholesterol acceptor remains to be determined, using BDS in this assay does appear to accurately reflect physiological cholesterol efflux. On the other hand, the CEC assay using HDL as the cholesterol acceptor is more representative of an estimation of cholesterol efflux ability per unit mass of HDL. While further study is needed to fine tune this method, especially with regards to the PEG precipitation method, our data suggest that this novel CEC assay could be widely used to estimate cardiovascular health in a quick and effective manner in clinical settings.

## Abbreviations

BDS: apolipoprotein B-100 depleted serum
CEC: cholesterol efflux capacity
ILG: immobilized liposome-bound gel beads
PEG: polyethylene glycol
RCT: reverse cholesterol transport
